# History of Mental Philosophy
*History of the Philosophy of Mind, embracing the opinions of all Writers on Mental Science, from the earliest to the present time. By Robert Blakey, Esq. In four vols. 1851.


**Published:** 1852-01-01

**Authors:** 


### Art. V.—HISTORY OF MENTAL PHILOSOPHY.*

Four large octavo volumes, of some five hundred pages each, on the subject of psychology, may, in themselves, be regarded as almost a literary phenomenon in this country. In Germany, and even in France, this subject has commanded a teeming press; while our greater propensity to physical pursuits in general, than to intellectual speculations, has prevented the birth among us even of an adequate history of a subject which lies at the foundation of the philosophy of all the moral sciences, not excluding even religion itself. At all events, it cannot now be said that we have not a history of philosophy in English, of goodly bulk and proportions—ushered into the world, withal, under auspices no less royal and popular than those of the Queen's consort; for to Prince Albert are these volumes dedicated, and with his Royal Highness's permission.

While this work professes to be a "History of Metaphysical Philosophy," and nothing more, the author has also given dissertations, in several places, on some of the elementary topics relating to psychology, unconnected with the course of simple chronological narration. In this method he has followed one or more recent writers on some branches of the same subject; but, like them, we think he would have done better to limit himself to the history of the subject; for we do not deem these digressions, by any means, the most successful parts of the work; and as Mr. Blakey has chosen to adopt the order of time, rather than of schools or of development, the method of mixing original matter with the history appears more interruptive of the thread of the narration than it might have been, had a different order than the chronological been adopted. The writers to whom he is particularly indebted for his details are De Gerando, Cousin, Damiron, Brucker, Ritter, Michelet, Dugald Stewart, Stanley, Cudworth, Enfield, and Hallam.

In his "Introduction," our author justly observes, that mental philosophy, in some form or other, is a want of human nature, since everything centres in mind. Even the *positive* philosopher, by which term the cultivator of natural science is denominated by the Germans and the French, is not in a position to mould his own facts, without the virtual recognition of principles which have a close relationship with mental science. We might exemplify this remark by a reference to the general principle of induction; for whenever the induction is not absolutely perfect, (as it is in the case of Aristotle's inductive syllogism,) by

* History of the Philosophy of Mind, embracing the opinions of all Writers on Mental Science, from the earliest to the present time. By Robert Blakey, Esq. In four vols. 1851.

means of the actual enumeration of all the particulars which go to constitute the general principle, we are obliged to proceed on the ground of that law of our mental constitution by which we cannot help believing that like causes produce like effects. But especially are three great divisions of theoretic knowledge allied to the psychological analysis—namely, morals, politics, and religion : for, without a close acquaintance with the mental functions and operations, little can be done in reducing either of these important branches of study to anything like a system. Freedom, duty, responsibility, obligation, conscience, moral actions, rewards, punishments,—all involve mental phenomena which require a strict analysis, in order to obtain a satisfactory basis for any proposed theory, ethical, political, or religious. He would certainly be a sorry moralist, jurisconsult, or divine, who should fail to seek the basis of ethics, jurisprudence, and theology, in the constitution of the human mind; although, no doubt, many have erred in not recognising this principle, whom it has most nearly concerned.

It is a fact which might be worth tracing to its causes, did space allow, that psychological studies have, in England, been comparatively so little cultivated for a century past. In Scotland they have obtained much more attention. Psychology can hardly be said to have formed any portion of academical instruction in this country, till the rise of the London University, in 1828, when it was made a part of the *curriculum*, as in Scotland, by the first Council. The change by which the original University became University College, and a new body was created by the government as a board of examiners, to be called the University of London, has proved anything but favourable and encouraging to psychological studies. The reason is, that mathematics pure and applied, and classical literature, constitute almost the entire arena in which honours and emoluments are to be obtained in the first liberal university which has been established in England. In Germany, psychological philosophy has been a marked feature of the educational literature of the country. It seems peculiarly characteristic of the genius of the German mind : hence, it has entered very essentially into the academical systems, under the head of psychology, metaphysic, logic, ethic, and æsthetic. Again, in the colleges of France, the candidate for the degree of *bachelier-ès-lettres* includes among his studies metaphysic, logic, and morals. In Trinity college, Dublin, "logic, the philosophy of the mind, and moral philosophy," form a considerable portion of the *curriculum*. The same may be said, especially of the two latter subjects, as regards the Scottish universities. In the system of our ancient English universities, the philosophy of the mental department of human nature has not formed one of the most prominent studies : the elements of logic, the ancient philosophical treatises

"Locke's Essay," and "Paley's Moral Philosophy," however, have entered, more or less, either optionally, or otherwise, into some of the examinations.

The university of London has included in the examination for the degree of B.A. a part of "Whately's Logic," a part of "Paley's Moral and Political Philosophy," and "Butler's Three Sermons on Human Nature." In this university the degree of M.A. may be taken in three different ways, which are open to the choice of the student; one way being by examination in "logic, moral philosophy, philosophy of the mind, political philosophy, and political economy." The degree of LL.B. involves examination in Bentham's "Theory of Morals and Legislation." Any candidates for the degree of M.D., who may not have previously graduated in arts, in London or elsewhere, are examined in the "elements of intellectual philosophy, logic, and moral philosophy." The above regulations may be seen in the London University calendar.

As the tendency is (as it ought to be) to demand a full general education for physicians, as preparatory to that which is strictly professional, there is little doubt that, ere long, all will previously graduate in arts; so that this philosophical examination for the degree of M.D. will soon become obsolete. Indeed, it is not easy to see on what consistent principle the degree of B.A. should, in this case, be a substitute for the examination in "intellectual philosophy;" since no "intellectual philosophy" (proper) is demanded for the B.A. degree, or its honours. The legal degree, again, only touches the subject of psychology in a very limited manner, and on the side of ethics. The examination for the B.A. degree demands but little logic, an acquaintance with only two writers on ethics, and no general philosophy of the mind. The students who have passed on to the degree of M.A. have hitherto been few in number, as compared with those who have taken the B.A. degree; a result not surprising, as many students are hurried to business and the professions at an early age. The calendar does not indicate the three distinctions among those who have passed, in the examination for the master's degree; but it is probable that not more than one-third of the whole number who have taken this degree, have graduated for the *moral sciences*.

On the whole, the curriculum of the university of London, as at present arranged, will be found giving, practically, but a small amount of encouragement to "philosophy," as this term is understood in the universities of the Continent of Europe and of the sister kingdoms. The graduation for the baccalaureat would seem to be the proper occasion for insuring that the student shall possess a competent acquaintance with the subject, by having prepared himself for examination in the

"History of Philosophy." This would go far to secure that the bulk of the graduates shall not be ignorant of inquiries which have a close bearing on everything that is related to the highest interests of man. Nor would it follow, any more than it now does, that the student should be committed to any one *particular school* of philosophy: for the hand may not yet have appeared which shall have power to grasp conflicting elements, and satisfactorily to combine into one harmonious system whatever is valuable, because true, in the schools of antiquity, and in those of the modern transcendentalism, with our own prevailing more experimental and inductive methods. As matters now are, it certainly appears, to any one who judges from the actual requirements stated in the first seventy pages of the calendar, that the student may go forth into society as the representative of the university of London, by its degree being attached to his name—nay, that it is even possible for him to take, in succession, every degree which it confers, in arts, law, and medicine, and with honours too, and yet to escape any historical acquaintance with the philosophical speculations of such writers as Bacon, Hobbes, Descartes, Leibnitz, Locke, Berkeley, Hume, Reid, Stewart, Brown, Kant, Schelling, Hegel, Cousin, and Jouffroy.

Our author, in justly vindicating for the philosophy of mind a greater degree of attention than it has yet gained, as an academical pursuit, in England, closes his introduction by a few general remarks by way of illustrating what he thinks may be admitted with certainty in relation to the subject. Among these is the observation that the separate and distinct characters of matter and mind are established by the historical statements and details of all ages; and that we have here a "solemn unity of universal assent, which no hardihood of assertion can deny, nor captious sophistry gainsay." We have no desire to dispute the general doctrine of immaterialism, but we think it far more philosophical to treat psychology as mainly a *phenomenal* science; a science, we mean, which inquires into the operations of the mind, and the functions to which they belong. The ontological question regarding materialism and immaterialism, we consider as at least an after consideration, so far as mere human philosophy is concerned, and from its transcendental character as rather belonging to the sequel than to the outset of a course of mental philosophy. But however this may be, we simply state a fact when we say that Mr. Blakey's assertion is here much too general; for certainly we are not authorized to say that the earliest Greek notions on mind were uniformly immaterialistic. The speculations of Thales, Anaximenes, Diogenes of Apollonia, and the Epicureans, respecting the soul appear to have been clearly materialistic; and it is doubtful whether to this list we should not add the Stoics.

It was natural that speculative thinkers should, at all periods of civilization, feel a high interest in the opinions of those who had preceded them. Accordingly the history of philosophy was coeval with philosophy itself. It could hardly fail to blend with all the original matter which the great men of antiquity have recorded, and which has still been preserved to us as a costly treasure amid the wrecks of time. Hence Plato and Aristotle have furnished us with invaluable materials of this kind; Cicero, the prince of the Roman literati, has handed down many dogmas of the Greeks by way of illustrating his own eclecticism; and we are indebted to the laborious Germans for a complete digest of passages from the great orator relative to philosophy, which work was published in Berlin, near the close of the last century. We may add Xenophon and Lucretius as great authorities for those parts of the history of philosophy which came under their more immediate notice; also Diogenes Laertius, Seneca, and Sextus Empiricus. Galen, and several of the early fathers of the Christian church, have also been contributors to the same general store. In the fourth century, Eunapius and Stobæus laboured in the same field; and in the sixth century, Hesychius gave to the world a biographical abridgment chiefly from Diogenes Laertius.

The revival of learning after the dark ages, brought a revived attention to philosophical speculations; and, in the fifteenth century, Burley published his "Lives of the Philosophers," soon after the art of printing had gained a footing in England. In Italy, Ficinus and Pomponius produced a new interest, among their countrymen, in the writings of Plato and Aristotle. Other historians of philosophy followed; till, at last, in recent times, the history of philosophy has formed one of the grand burthens of the press on the continent, but especially in Germany.

Our author begins with the mental philosophy of Greece, which occupies nearly half of the first volume. But the work, though likely to be not uninteresting to the general reader, is of too popular a character for very close dealing with the Greeks; the philosophical critic being for the most part pretty much sunk in the historian. There is also, frequently, a want of keeping as to the amount of matter awarded to the several writers. Thus, we should have expected that, in so voluminous a work, Plato would hardly have been dismissed with a dozen pages; and that Aristotle's recondite and laborious speculations, in his "Metaphysic" and his "Organon," would have been thought to require rather more than some five and twenty pages to do him anything like justice.

After treating of the Roman, the Sceptical, the Indian, the Neo-Platonistic, and the Patristic schools of philosophy, the author gives to the reader some of his own psychological views; more particularly on the doctrine of "distinct faculties or powers of the mind." He appears, for instance, to object to the ordinary distinction between "judgment and imagination." The author maintains that the only difference between the two lies in the circumstance, that "the ideas the mind is employed about are true in the one case and false in the other;" in other words, that "real and fictitious representations constitute the only difference between these two mental powers." Our space will not allow us to digress at length on any one point of the author's original opinions; but we do not hesitate to say, that to us, apart from all the ambiguity which we are aware may attach to the language employed, his views on this subject are by no means satisfactory. There is surely a difference to consciousness in a reverie which may flit through the mind in a waking dream, and a case in which the mind apprehends or denies a distinct relation between a subject and a predicate. The former we, as everybody else, should call an exercise of imagination; the latter an exercise of judgment. With, at least, equal infelicity, as seems to us, Mr. Blakey denies all sort of *analysis* to thought. He says, "If they (thoughts) can be analyzed, they can be subdivided; and what is capable of subdivision may be divided in *infinitum*. Then, if thoughts be infinitely divisible, they must be infinitely extended, and what is infinitely divisible and extended can have no elementary parts; consequently, thoughts must be nothing at all. What a fine doctrine for the sceptics!" This may be ingenious; but we think it not more conclusive than a familiar pseudo-sorites, which is sometimes given in books of logic, as an example of the fallacies; "France is the finest country in Europe; Paris is the finest city in France; this salon is the finest room in Paris; my uncle is the finest man in the salon; therefore my uncle is the finest man in France."

The chapter on the 'Metaphysical Disquisitions of the Ancient Fathers of the Church," suggests a field for most interesting matter. Their opinions are the more worthy of attention, because the establishment of Christianity introduced a new element into philosophical inquiries. It is impossible to take any definite views of the Christian Scriptures, so as to admit them to have an historical validity, without feeling it compulsory on reason to take into account their utterances, whenever they speak on any subjects that are closely blended with the philosophical speculations which have characterized civilized society from the earliest times, and always will continue to be a large element in the literature of nations. Not a few of the Christian fathers, moreover, were considerable adepts in the study of philosophical antiquity. Justin Martyr, for instance, had paid particular attention to the doctrines of the Greek schools, especially those of Plato and Aristotle. Eusebius tells us, that Justin wrote a work on the nature of the soul of man, in which he gave a digest of ancient opinions; but no such work is extant. Tatian's notions were somewhat mystical; for he maintained that there are, in all *good* men, two principles—the soul and the understanding, or *logos*. We find a similar tendency to mysticism in many of the fathers; and as in theology, so in philosophy, however valuable as witnesses, they are often very indifferent guides. Origen and others held the supposition of a metempsychosis, a dogma found both among the Gnostics and the Neo-Platonists. Not a few of the ancient Fathers, however, either opposed the study of profane philosophy altogether, or held strong prejudices against it. Among these were Hermes, Tertullian, Arnobius, Irenæus, and Lactantius. We have, in the sequel, a useful collection of the opinions of some of the Christian fathers, "as to their conceptions of free-will,"—a subject which is still debated as a controverted point by those who attempt to reduce the transcendental part of theology to system, and are not content with its practical bearing on man's actions, in which respect it is very plain. The views of many of the Fathers on this subject are given in actual quotations from their works; among some of which we might trace some of the same casts of thought which have prevailed in modern times among the respective advocates of the libertarian and the necessarian schemes, as advocated, for instance, by Dr. Samuel Clarke on the one hand, and Jonathan Edwards on the other.

The thirty-sixth and thirty-seventh chapters contain, "Remarks on the Faculties of the Mind," and on what may be urged against their individual existence, nature, and operations." Our author does not begin, as he might very properly have done, with telling us what he would understand by "faculties of the mind," and "distinct faculties;" but he argues strenuously "against distinct faculties or powers of the mind," as though this doctrine were opposed to the "absolute unity or singleness of the mind of man." We do not see how this need be the case, any more than a distinction in the animal and organic functions of man's body opposes the idea of one corporeal life. Mr. Blakey maintains, for instance, as we have seen, that there is no difference between judgment and imagination..

Bating what may be due here to the ambiguity of terms, which ought always to be carefully thought of in all metaphysical questions, we are of opinion that the whole of this discussion is by no means satisfactory: for surely the main objection to the common view of distinct faculties must fall to the ground when we take almost any example of this doctrine. For example—has not man the power of thinking, and has he not the power of feeling?—of thinking ideas and trains of them, and of feeling bodily sensations—and, in a different way, of feeling emotions, such as joy or grief? May we not say that the phenomena of thought, of sensation, of emotion, are very obviously distinct to consciousness, and that it is quite conceivable that man might have had the first of these without the others? and wherein consists the impropriety of supposing that these varied functions belong to one and the same being? Indeed, is not this a fact? and why should we not speak of these revelations of the constitution of the mind as the results of different powers? We can hardly conceive of any language less likely to be misunderstood, provided we do not mean by it that the phenomena in question always necessarily exist apart, which they certainly do not; and which the doctrine of "different faculties" by no means needs to be regarded as implying.

In the chapter on "Saxon Metaphysics," we have quotations from Alfred the Great, Alcuinus, and Bede. It is interesting to know what were the meditations which occupied the leisure hours of the greatest of our monarchs. The following speculations on "chance and freedom," by King Alfred, are from "Turner's Anglo-Saxon History:"—

"It is nought when men say anything happens by chance; because everything comes from some other things or causes, therefore, it has not happened from chance; but if it come not from anything, then it would have occurred from chance. Then said I, whence first came the name? then quoth he, my darling Aristotle maintained it in the book that is called 'Phisica.' Then said I, how does he explain it? He answered, men said formerly, when anything happened to them unexpectedly, that this was by chance. As if any one should dig the earth, and find there a treasure of gold, and should then say that this happened by chance, but yet I know that if the digger had not dug into the earth, and no man before had hidden the gold there, he would by no means have found it. Therefore it was not found by chance.

"On the freedom of the will, I would ask thee, whether we have any freedom or any power, what we should do, or what we should not do? or does the divine pre-ordination or fate compel us to that which we wish? then, said he, we must have power. There is no rational creature which has not freedom. He that hath reason, may judge and discriminate what he should will, and what he should shun; and every man hath this freedom, that he knows what he should will and what he should not will. Yet, all rational creatures have not a like freedôm. Angels have right judgment, and good will, and all that they desire they obtain very easily, because they wish nothing wrong. But no creature hath freedom and reason except angels and men. Men have always freedom, and the more of it as they lead their minds towards divine things. But they have less freedom when they incline their minds near to this world's wealth and honours. They have no freedom when they themselves subject their own wills to the vices; but so soon as they turn away their mind from good, they are blinded with unwiseness."

The second volume, after introducing us to the scholastic metaphysicans, devotes very properly a few pages to Lord Bacon; and it is justly (so far at least, as our own country is concerned) observed by the author, that after the time of this truly distinguished man, the whole aspect of metaphysical philosophy was altered, and his genius exercised a most beneficial influence on subsequent speculations of this kind; so that we have scarcely an instance, since his day, of a single eminent man falling back into the old scholastic mode of treating speculative philosophy. Dugald Stewart has well observed, that although Bacon, on some occasions, assumes the existence of "animal spirits" as the medium of communication between soul and body, which dogma was then universal among the learned, yet the theory is commonly so alluded to by this illustrious man as that the facts of human nature can easily be detached from it; and as to the scholastic questions relating to the nature or essence of mind, whether it be extended or unextended, whether it have any relation to space or to time, or whether, as some maintained, it exists in *every ubi*, but in *no place*, Bacon has passed over these questions with "silent contempt," and as Stewart thinks, has "probably contributed not less effectually to bring them into general discredit by this indirect intimation of his own opinion, than if he had descended to the ungrateful task of exposing their absurdity." Bacon precisely distinguished between those ontological inquiries which the schoolmen vainly pursued, and which, with as little success, and quite as paradoxically, have been ardently carried on by the German philosophers—and those more modest psychological investigations which content themselves with discovering and registering the phenomena and the functions of man as an intelligent and moral being. Though Bacon did not forbid more speculative disquisitions, it is evident that he placed them on a different footing from that inductive psychology, which so much more harmonized with his own physical method. On the limits and boundaries of human knowledge, he makes the following observations:—

"For human knowledge which concerns the mind, it hath two parts; the one that inquireth of the substance or nature of the soul or mind, the other that inquireth of the faculties or functions thereof. Unto the first of these, the considerations of the original of the soul, whether it be native or adventative, and how far it is exempted from laws of matter, and of the immortality thereof, and many other points do appertain: which have been not more laboriously inquired than variously reported; so as the travail thereon taken seemeth to have been rather a maze than a way. But although I am of opinion that this knowledge may be more really and soundly inquired even in nature than it hath yet been, yet I hold that in the end it must be bounded by religion, or else it will be subject to deceit and delusion; for as the substance of the soul in the creation was not extracted out of the mass of heaven and earth by the benediction of a 'producat,' but was immediately inspired from God; so it is not possible that it should be otherwise than by accident, subject to the laws of heaven and earth, which are the subject of philosophy; and therefore the true knowledge of the nature and state of the soul, must come by the same inspiration that gives the substance."

In regard to final causes, Bacon appears to have laid but little stress, though it is certain that a sober investigation into them, under the careful auspices of his own method, has not been without its effect on science: witness the discovery of the circulation of the blood, and that of achromatic telescopes, for example. Probably Bacon was repelled from much encouraging this path of inquiry by the vagaries of some of the schoolmen, and the fanciful analogies and hypotheses in which it had, in his time, so long been the fashion to indulge. Still he was far from being insensible that it might lead to truth.

"The search into first causes is barren, and like a virgin consecrated to God, it brings forth nothing. (This) second part of metaphysics I object to; not as a speculation which ought to be neglected, but as one which has, in general, been very improperly regarded as a branch of physics. If this were merely a fault of method, I should not be disposed to lay great stress upon it. But, in this instance, a disregard of method has occasioned the most fatal consequences to philosophy; inasmuch as the consideration of first causes in physics has supplanted and banished the study of physical causes; the fancy amusing itself with illusory explanations derived from the former, and misleading the curiosity from a steady prosecution of the latter. . . . . . . . I would not, however, be understood by these observations, to insinuate that the final causes just mentioned may not be found in truth, and in a metaphysical view, extremely worthy of attention; but only that when such disquisitions invade and overrun the appropriate province of physics, they are likely to lay waste and ruin that department of knowledge."

After Descartes, Spinoza has very properly some thirty pages allotted to him; for the bearing of his opinions on some of the later German speculations is great and obvious. Our author states that this extraordinary man was expelled from the synagogue at Amsterdam (being a Jew) for contumacy to his parents; there is no doubt, however, we apprehend, that his real offence was the freedom of his opinions. There is something tragical and terrible in the description which Mr. Lewes, in his "Biographical History of Philosophy," briefly gives of this event:—

"The day of excommunication at length arrived, and a vast concourse of Jews assembled to witness the awful ceremony. It began by the solemn and silent lighting of a quantity of black wax candles, and by opening the tabernacle wherein were deposited the books of the law of Moses. Thus were the dim imaginations of the faithful prepared for all the horror of the scene. Morteira, the ancient friend and master, now the fiercest enemy of the condemned, was to order the execution. He stood there, pained, but implacable; the people fixed their eager eyes upon him. High above, the chanter rose and chanted forth, in loud lugubrious tones, the words of execration; while, from the opposite side, another mingled with these curses the thrilling sounds of the trumpet; and now the black candles were reversed, and were made to melt drop by drop, into a huge vessel filled with blood! This spectacle—a symbol of the most terrible faith—made the whole assembly shudder; and when the final '*anathema maranatha!*' was uttered, and the lights all suddenly immersed in the blood, a cry of religious horror and execration burst from all; and in that solemn darkness, and to those solemn curses, they shouted—'Amen! Amen!' Thus was the young truth-seeker expelled from his community, and his friends and relations forbidden to hold intercourse with him."

Perhaps our author hardly does justice to Spinoza as a man. He appears to have been very amiable, his personal character was without blame, and none can doubt the sincerity with which he held his opinions; which, indeed, was proved by his being willing to suffer for them. If devout expressions respecting the Deity, and speaking of "loving him," mean anything, Spinoza was not himself an atheist; although the iron necessity to which he considered the Deity subjected, and the inadequate ideas he had of divine personality and will, were no doubt calculated to lead to atheism. That Spinoza's pantheism has given a tone to German speculation, since the time of Kant, (who, whatever his idealism, stopped short far enough from pantheism,) we have never heard questioned. Our author, however, does not allude to this influence. No doubt the pantheisms of Fichte and Hegel are different from that of Spinoza. His pantheism we might describe, in German phrase, as objective, realistic, and plastic. He does not confound the deity with the *ego*, but he understands the Deity to be the substratum, or immanent cause of the *ego*. God is a real being, not an idea. He manifests himself by a plastic energy in bodies and in minds, which are respectively portions of his infinite attributes of extension and thought. Fichte's pantheism, on the other hand, is an *ich-lehre*, or doctrine of the *ego*, in which the Deity is identified with the moral order of the universe, as conceived by the free activity of the *ego*. This Fichtean pantheism does not distinguish God from the operations of the human mind; and as it assumes that matter does not exist, it is a subjective, idealistic pantheism. Hegel's system, again, was an absolute pantheistic idealism: thought, with him, being the only true and real existence, and the Deity being nothing more than a development of thought in human consciousness. Schelling's pantheism the most nearly resembles that of Spinoza, being *realistic*, or maintaining the real existence of Deity, in distinction from the notion that God is merely an ideal being. It is also *objective*, as not making the deity dependent on the development of the *ego*. It is, indeed, more objective with respect to the *finite* than Spinozism itself; for the latter made bodies and minds only modes of the infinite: Schelling, on the contrary, holds that the finite is not so involved in the infinite as to lose its own real existence.

We pass over many distinguished names, to devote a few lines to Mr. Blakey's estimate of Locke. He regards the "most vulnerable point of Locke's system" to be his doctrine of innate ideas. Perhaps it is so—unless the extraordinary jumble which Locke has made of "*personal identity*," be even a still greater fault. For, on this latter point, Locke certainly either talks what amounts to absurdity, saying that our personal identity depends on memory, or else, by using the term person in a sense of which he gives little or no warning, he departs unwarrantably from all the conventionalities of language. On the subject of innate ideas, our author remarks:—

"The weakness of Locke's arguments, however, appears to me to lie more in his language than in his proofs themselves. He does not deny that men have a certain *innate capacity* to recognise truths of the most abstract form and nature; but he affirms that the mind is not born with these truths put into the *shape of axioms*. There must be previous sensations experienced before truths of such a nature can be appreciated by the mind. Self-evident propositions—such, *e. g.*, as that it is impossible for the same thing to be and not to be, or that whole is greater than its part—cannot be known by children or savages. This is quite true; but the observation does not meet the merits of the question. Children or savages may not comprehend these axioms, when put into the formal drapery of logical terms; but all their reasonings and movements of life, are grounded upon a full and complete recognition of these and similar abstract forms of thought. Experience may precede, but it does not create those general truths. They are part and parcel of the mind itself. We are not born lisping abstract axioms; but they are immediately recognised by every sane mind, the moment that the terms in which they are involved are sufficiently understood. There could be no general or scientific truth unless these elementary principles of thought and reasoning were universally diffused among our race.

"Now, it is very doubtful that Locke ever for a moment thought of denying the *innate materials of thought* out of which those formal axioms are derived. He says he maintains the *capacity* to know abstract truths, and that this may be considered in a certain point of view as *innate*. This brings the dispute within a narrow compass."

"I allow," says Locke, "that there is in the mind an innate capacity to form and conceive certain universal propositions, but I deny that men are born with these formal axioms ready framed in their understandings. His opponents reply, we allow these axioms are not clothed in a logical dress in the infant or savage man, but that these minds contain the rudiments, or germs, or elements of these axioms, as a part of their spiritual or mental nature. This appears to me the sum or substance of the difference between Mr. Locke and his critics, on this part of the question of innate ideas. If there be a real difference, it is one resting solely on the different terms in which both parties express themselves." Vol. ii. p. 469.

We doubt much whether the author has, in this criticism, quite sounded the depth of Locke's delinquency in the matter of "innate ideas." He not only denies that any ideas, or any truths are *born with us*, which neither Descartes nor Leibnitz ever pretended; but the real theoretic point in which, as we conceive, he differed from those illustrious writers was that he wished to bring all ideas and all truths, in a similar manner, without distinction, under the general category of *experience*: for in his chapters on "Innate Principles," after rejecting the doctrine of Descartes and Leibnitz, he proceeds to trace the "steps by which the mind attains several truths;" and he says that the mind grows familiar by degrees with the ideas let in by the senses; and afterwards the mind proceeding further abstracts them, and by degrees learns the use of general names. He asserts that our knowledge of the truths in question is about ideas not innate but "*acquired*." A child, he adds, must stay till time and observation have acquainted him with them. "This evasion of general assent fails, and leaves no difference between those supposed innate and other truths that are afterwards acquired and learned."

Now this sort of language, and a good deal more like it, appears to us plainly to show that Locke did not mean to make any distinction between such a truth as "every change in the universe must have a cause," and the following—"all horned animals have cloven feet." We doubt not that Locke would have said, as we have heard affirmed now-a-days, that both these truths stand on the common ground of experience. The fact is that Locke made no distinction, such as that of Leibnitz and Kant, and which the latter especially has so elaborately and clearly pointed out, between truths that are learned by habit or experience, and truths which are grasped and fully recognised by the mind the very first time that any example of their general formula (so to say) presents itself to the mind. The earlier German school has most clearly exhibited this difference—the difference there is between truths which cannot be said to be fully learned and appreciated until many instances in illustration of them have occurred to the mind—and those truths which never, during the whole course of life, appear one whit more certain, from the very first instant when a single illustration of them occurred. In other words, Locke, though not in terms, yet really, set himself to annihilate theoretically the distinction between inductive generalizations and certain *à priori* and self-evident truths. To say that Locke admitted a certain *innate capacity* to recognise abstract truths, while he denied that men are born with formal axioms in their understandings, is saying but little to the point in question. Yet our author thinks that the sum and substance of the difference between Locke and his opponents consists "solely in the different terms in which both parties express themselves." Surely neither Descartes, nor Leibnitz, nor Hume, nor Reid, nor Kant, would have admitted this solution. The fact appears to us to be, that Locke was wrong in the outset in arguing as though the Cartesians imagined that infants were born with ideas or propositions in their minds. He was wrong in seeming to deny all intellectual and moral instinct, or intuition, in theory; although no one can more clearly admit them both, virtually, when he had once lost sight of the controversy. An example occurs in what he, in one place, says of religious obligation, when he affirms to the effect, that if a man has the idea in his mind of an infinitely wise, powerful, and good being, who is a benefactor to a limited and dependent being, he can no more avoid perceiving that the limited dependent being ought to worship and reverence the Great Benefactor, than he can help seeing the light of the sun at noon-day.

Locke's essay would have better sustained the credit of the author, if he had altogether omitted the disquisition on "innate ideas and principles." Indeed, in his abridgment of it, published in Le Clerc's journal, this part is wanting, as he "thought it best, he tells us, to omit from his short abridgment all the preliminary disputes which were noticed, in order to destroy the prejudices of certain philosophers."

Locke's unsatisfactory treatment of this part of his subject amounts to what we may term almost a gratuitous damage to his Essay: for that he really did admit, perhaps unconscious of inconsistency, what he appears to every one who reads him to argue against, cannot be doubted. Witness, for example, the following passage, which proves, that notwithstanding his apparent theoretical contradiction of *à priori* ideas and truths, he contended for that necessity and universality which are their chief characteristics.

"There is one sort of propositions concerning the existence of anything answerable to such an idea—as, having the idea of an elephant, phœnix, motion, or angle in my mind, the first and natural inquiry is whether such a thing does anywhere exist? and this knowledge is only of particulars. No existence of anything without us, except God, can be certainly known further than our senses inform us."

"There is another sort of proposition, wherein is expressed the agreement or disagreement of our abstract ideas and their dependence on one another. Such propositions may be universal and certain: so, having the idea of God and myself, of fear and obedience, I cannot but be sure that God is to be feared and obeyed by me: and this proposition will be certain concerning man in general, if I have made an abstract idea of such species whereof I am one particular. But yet this proposition, how certain soever, that men ought to fear and obey God, proves not to me the existence of man in the world, but will be sure of all such creatures wherever they do exist: which certainty of such general proposition depends on the agreement or disagreement to be discovered in those abstract ideas. In the former case our knowledge is the consequence of the existence of things producing ideas in our minds by our senses; in the latter, knowledge is the consequence of the ideas (be they what they will) that are in our minds, producing there general certain propositions.

"Many of these are called *æternæ veritates,* and all of them indeed are so; not from being written in the minds of all men, or that they were any of them propositions in any one's mind till he, having got the abstract ideas, joined or separated them by affirmation or negation." (Descartes, Leibnitz, and Kant himself, would readily, in various phraseology, have admitted this.) "But whenever we can suppose such a creature as man is endowed with such faculties, and thereby furnished with such ideas as we have, we must conclude he must needs, when he applies his thoughts to the consideration of his ideas, know the truth of certain propositions that will arise from the agreement or disagreement which he will perceive in his own ideas. Such propositions, therefore, are called eternal truths, not because they are eternal propositions actually formed and antecedent to the understanding that makes them, nor because they are imprinted on the mind from any patterns that are anywhere of them out of the mind and existed before; but because being once made about abstract ideas so as to be true, they will, whenever they can be supposed to be made again at any time by a mind having those ideas, always actually be true."—Essay, book iv. ch. 2.

Our author has justly assigned a place in his work to Dr. Samuel Clarke. We are not, however, quite satisfied with the account given of the basis of this distinguished writer's argument for the divine existence; which is said to be the following statement from Newton's "Principia:"—

"God is eternal and infinite, omnipotent and omniscient; that is, he endures from everlasting to everlasting, and is present from infinity to infinity. He is not eternity and infinity, but eternal and infinite. He is not duration or space, but he endures and is present. He endures always, and is present everywhere; and by existing always and everywhere constitutes duration and space."

"Upon this foundation," says the author, "Dr. Clarke endeavoured to raise his *à priori* argument for the existence of a Deity." Surely not so: the foundation of Clarke's "Demonstration of the Being and Attributes of God," was the following: "It is evident that something does now actually exist." (*e. g.* we ourselves, the universe, &c.) If something has not existed from eternity, the things which now are must have risen absolutely from nothing, and without any producing cause; we are, therefore certain something has existed from eternity."

The truth is not exactly that the learned have "abandoned" Clarke's line of argument, which is still allowed to contain some very beautiful trains of *à priori* reasoning; but trains which follow on the previous admission of a Deity. The fault of Clarke's argument consists in its claim to be an *à priori* or mathematical demonstration of the divine existence (though still with some inconsistent admissions), whereas, at the outset a matter of fact is assumed, and no matter of fact is capable of mathematical proof. This matter of fact is that something exists, and, it is affirmed, that it can only be accounted for on the supposition of a Deity. The account of Clarke would have been improved, if an analysis had been given of the celebrated controvery between him and Leibnitz, but it is only alluded to under either names.

About twenty pages are devoted to Dr. Reid, who may be regarded as the main founder of the Scottish school of psychology. We must limit our remarks on the author's notice of this eminent philosophical writer to one point. He says, on the subject of Reid's realism as opposed to Berkeley's idealism, that—

"Reid's arguments against those who deny the existence of matter are certainly very weak and defective. He lays down the position himself, that preception is entirely an art of the mind; so that he does, in substance only, affirm the same thing as Berkeley and Hume do. Both assert that we cannot go beyond our own consciousness, and therefore can never know things *per se;* but they never call in question the common sense belief that matter exists externally. The grand argument, therefore, used by Reid, for the existence of an external world, is founded on the irresistible belief which arises from preception and memory. That this belief is universal and influential, no one can question, not even the sceptics themselves; but it may still be affirmed that this is not *proving* the existence of anything beyond the existence of mere perception and memory."

Unquestionably, Berkeley *did* deny that "matter exists externally;" and Reid himself did not profess to *prove* the existence of matter, any more than Descartes professed to prove the existence of self. He considered it a primary element of belief; and thus did Reid consider the existence of a non-*ego* without us. He says—

"The belief of a material world declines the tribunal of reason, and laughs at all the artillery of the logician. Reason itself must stoop to its orders. Therefore, since we cannot get rid of the vulgar notion and belief of an external world, it were better to make a virtue of necessity, and to reconcile our reason to it as well as we can."

No doubt Reid maintained the doctrine of a material world, on the principle that we just cannot help believing it; proof he brought none, and pretended to none. Whether he erred in limiting the mental energy to a mere consciousness of the *ego* and its operations—denying it any immediate cognizance of the *non-ego*—is another question. Even this immediate cognizance, if it exists, can hardly be called proof. It is beyond proof—it is intuition.

The middle of this third volume brings us to Kant, the chief of the more modern German school. Some of our readers, who have not read any German metaphysics, may be curious to know what was the origin and design of the speculations of the great author of the critical idealism. In his "Prolegomena to all Future Metaphysic," he says—

"My intention is to convince all who occupy themselves with metaphysic, that it is necessary first to settle the question whether metaphysic be possible. Since the essays of Locke and Leibnitz, or rather since the origin of metaphysic, no event has occurred so calculated to decide the fate of this science as the attack made upon it by Hume, who took up a single but important conception, that of cause and effect; consequently the derived conceptions of power, action, &c., and challenged reason, which holds it up as its own produce, to say by what right it concludes that one thing may be so constituted, that if it be given something else must necessarily be inferred, for this is the meaning of the conception of a cause. He proved beyond contradiction, that it is quite impossible for reason to discover in the conceptions themselves any necessary connexion, since we cannot see why, because something is, something else must necessarily also be; and consequently we are at a loss to know how the conception of such a connexion, *à priori*, can have arisen. Hence, he concluded that reason entirely deluded itself with this conception, falsely considering it as its own offspring, while in fact it is nothing more than a bastard of the imagination; which, pregnant by experience, has brought certain representations under the law of association, and substituted a sort of subjective necessity—namely, habit—for an objective one founded upon real knowledge. He concluded, therefore, that reason had no faculty to think such connexions, even generally, because its conceptions would in that case be mere fiction, and all its pretended knowledge *à priori* nothing but a false value given to common experience. In other words, that no such science as metaphysic is at all possible. However hasty and incorrect his inference was, it was grounded at least upon investigation. However, no one understood Hume's intention. His opponents—Reid, Oswald, Beattie, and Priestly,—missed the point of his question; always admitting, as a matter of course, the very thing which he doubted, and proving with vehemence that which it never entered his mind to doubt. The question was not, whether the conception of cause be indispensible to all knowledge of nature,—this Hume never doubted,—but whether this conception be *thought by reason à priori*, and whether it possess, on that account, an internal truth independently of all experience, and therefore a more extensive utility, not limited to objects of experience. This hint of David Hume was the circumstance that first disturbed my dogmatical (Wolfian) slumbers, and gave a new direction to my researches. I was far from listening to his inferences, which proceeded merely from his not representing to himself his problem in its whole extent, but investigating merely a part of it, the solution of which was impossible without a comprehensive view of the whole. I soon found that the idea of cause and effect is by no means the only one in which the understanding represents to itself a connexion of things *à priori*, but that the whole of metaphysic consists of nothing else. I endeavoured to ascertain their number; and having done this to my satisfaction, upon a single principle, I proceeded to the deduction of these conceptions. I now saw that they were not derived, as Hume supposed, from experience, but that they originated in the understanding itself."

These understanding conceptions (*verstandes-begriffe*) Kant made to be twelve in number, these being sub-categories of the general categories of quantity, quality, relation, and modality. We have here the whole basis of Kant's critical idealism as given in the *Kritik der reinen Vernunft*. But here we must pause, for even to make thus much wholly intelligible to the English student (and intelligent enough it is), would require a space which we can by no means afford. The above quotation occurs, in part, in the volumes before us; and it shews the main drift of the *Kritik der reinen Vernunft*. It was to ascertain the *à priori* elements of the understanding and reason; and throughout his great work, the acute though somewhat pedantic German philosopher rings perpetual changes on the above twelve categories. We do not imagine that, as our author seems to imply, if not to say, the question of human freedom was prominent in the mind of Kant when he sat down to frame his celebrated categories, suggested by Hume's one category of causation. It is true that one of his "Antinomies," in an advanced part of the *Kritik* of Reason, consists of an opposition of the "Thesis" which asserts free causes, to the "Antithesis" which denies them: but we have here little more than one illustration of Kant's doctrine of the powerlessness of reason in all cases which are not strictly within the bounds of experience. His discussion of the freedom of the will is reserved for another work, entitled Practical Reason (*Praktische Vernunft*), which Kant identifies, in fact, with moral feeling and principle, or the operation of conscience. In the attempt to expound Kant and other German philosophers, many Englishmen have no doubt failed; and we cannot promise the close student of Kant and his successors that he will find in these volumes the clue of Ariadne, by which to wend his way through the labyrinth of German metaphysics, and to conquer the formidable Minotaurs of that marvellous region of mysteries and shadows. Yet those who are willing to content themselves with a popular glance at these extraordinary speculations, having no time, and it may be no inclination for anything further, may here perhaps find what may in some measure gratify their curiosity. The author gives the following just statement of the peculiar characteristic of the German school:

"The German mode of philosophising is radically distinct from ours. We usually commence with analyzing mental feelings and faculties; with instituting inquiries into the *outward* manifestations of mind; and from these draw certain conclusions and inferences. Now this is a very humble and subordinate department of science in the estimation of the German. He has more lofty aspirations, and aims at doing greater things. He plunges into the deepest recesses of what he calls *himself*, his inward and living principle; and categorically demands to know the reasons why it is as it is, and why he is stimulated and goaded on to know the why and the wherefore of his own individual existence, as well as the existence of everything which surrounds him. He seizes hold of his own mind or consciousness, and compels it to submit to a peremptory interrogation and cross-examination. He does not trouble himself much about an external world, for his purpose is to dig a deep and firm foundation out of his own thinking principle. Here he seeks for the primitive truth—the *Urwahr*, or the absolutely and eternally true."

It is so unusual to meet with a lady metaphysician, that we are tempted to introduce to our readers Lady Mary Shepherd, authoress of an "Essay on the Relation of Cause and Effect," and also of "Essays on the Perception of an External Universe, and other subjects connected with the doctrine of Causation;" the former work being published in 1824, and the latter in 1827. We had previously heard of this lady, who has trodden in a path somewhat unbeaten by female footsteps; but not having happened to meet with her writings, we are glad of the professor's introducton to her acquaintance. He gives her credit for having written works which, "considered as the productions of a lady," are justly entitled to high praise. We are not sure that ladies generally feel much flattered by this somewhat left-handed commendation; but be this as it may, it appears that Lady Shepherd, actuated by the sincere conviction that the views of Hume, Reid, Stewart, and Brown, on causation, "led by an inevitable consequence to downright atheism," endeavoured to counteract them by availing herself of the press; and Mr. Blakey thinks that the views in question did much harm in Scotland, and that Lady Shepherd did much to remedy the evil.

"Every young man who came from the universities of Scotland, attempted to show off his subtilty and academic lore, by denying that there was an real causation in the world; all was mere imagination, and a piece of gross vulgar credulity. Her ladyship's efforts were therefore well-timed; and there is no doubt, but their influence was decided in giving a considerable check to these illogical and dangerous opinions."

We confess that, with some personal knowledge of the state of things in the Scottish universities towards the close of the period referred to, we cannot but think that the effect of the alleged doctrine of causation, as here stated, is somewhat exaggerated. And as to the doctrine itself, Dr. Thomas Brown, its most luminous and detailed expounder, was unquestionably a very sincere and devout theist; and in advocating his theory of causation, he believed, no doubt, that he was merely insisting on the fact of human ignorance respecting any tie which may bind together cause and effect. For example, we know that the fluoric acid dissolves flint; we know the fact, but the *modus operandi* we know not; and so of all cases. The general propositions which Lady Mary Shepherd proposes to establish, are the following :—

"1. That objects cannot begin their own existence; 2. That like objects must have like qualities; 3. That like causes must generate like effects; 4. That objects of which we have had no experience must resemble those of which we have had experience, for that the course of nature continues uniformly the same."

We are not aware that Dr. Brown would for a moment have disputed any one of these propositions, understood with such explanations of their meaning as it appears to us must have been intended by the fair authoress. Her ladyship's talent for abstract thinking may perhaps be better illustrated by the following quotation from her essays, and this independently of any criticism of her theory. The subject is the certainty of our own existence:—

"The idea of our own independent existence is generated by observing that the compound mass we term self can exist when we do not observe it; and we have thus the idea of our own existence, in that it needs must continue to exist when unperceived, as well as during the *sensation* of it when perceived. Besides, on this subject, as on every other, it is the causes for the *constant* effects (the objects whose union shall bear out similar results), to which there is a tacit reference as the true and continued existences in nature. Now the causes for the general powers of sensation cannot be the same as those for any particular sensation, and so must be independent of each; and indeed each sensation is always *felt* as an effect, 'as beginning to be;' therefore, what we allude to as *self*, is a continued existing capacity in nature (unknown, unperceived), fitted to revive when suspended in sleep, or otherwise, and to keep up during the periods of watchfulness the powers of life and consciousness, especially those which determine the union of memory with sense. For as sensation is interrupted, and is an *effect*, the original cause must be uninterrupted; and such an uninterrupted cause as is equal to keep up the life of the body, or mass deemed our own body, and to unite it under that form with the powers of memory and sense. Identity, therefore, has nothing to do with *sameness of particles,* but only has relation to those powers in nature (flowing from that continuous being, the God of Nature) which are capable of giving birth to that constant effect, the *sense of continuous existence;* which sense, when analyzed, is the union of the *ideas of memory* with the *impressions of present sense.* Should it be objected that the causes for such a union might be interrupted, then as these would 'begin their existences,' and would *only be effects,* the mind would go backwards till it reposed in some *uninterrupted* cause, and would consider such, and such only, as an independent capacity in nature, fitted to excite the union of memory with present sense, and as the complicate being *self,* which, when conscious, would take notice of its existence, and when unconscious (as in sound sleep), would exist *independently* of its own observatios."

In conclusion, we are free to admit that, although we do not consider this work to be all that could be desired in point of execution, it is nevertheless valuable as a sort of general guide and index to the leading opinions of the most celebrated writers on psychology in all ages. What we should like to see is, a history of philosophy written in the condensed, analytical, and discriminative manner, which makes Dugald Stewart's Dissertation on this subject so truly valuable. Such a work must probably treat the subject by a *rationale* of the several schools, rather than in the chronological detail adopted in these volumes. Yet they are often deficient in detail. For instance, Plato and Aristotle are dismissed far too summarily. Their metaphysics do not occupy twenty pages of the work, independently of the notice of Aristotle's logic. The account, also, of a name so important as that of Leibnitz might have been expected to be more copious in a work of such extent : and, by the way, the author's views of the science of logic we hold to be altogether erroneous, cashiering it, as he does, altogether, in a small work published on this subject, from the domain of the exact sciences. We have not been struck with those parts of the work which contain the author's original dissertations on the "Faculties of the Mind," the "Nature of Truth," the "Sublime and Beautiful," &c. They are not characterized, as appears to us, by that high critical acumen which is required in order to redeem a history of philosophy from the irrelevancy of original discussions, and we think that the work would have been improved by their omission, and by the substitution of a more elaborate digest of the opinions of some of the principal psychologists. As it is, however, it may do something, and we hope it will, to introduce the subject further among general readers; and the high moral tone of the work renders it unexceptionable in whatever quarter it may find its way.

